# Evaluation of a Specialized Yoga Program for Persons Admitted to a Complex Continuing Care Hospital: A Pilot Study

**DOI:** 10.1155/2016/6267879

**Published:** 2016-12-27

**Authors:** Kathryn Curtis, Kerry Kuluski, Gitte Bechsgaard, Jennifer Ridgway, Joel Katz

**Affiliations:** ^1^Department of Psychology, Faculty of Health, York University, 4700 Keele St., Toronto, ON, Canada M3J 1P3; ^2^Lunenfeld-Tanenbaum Research Institute, Sinai Health System, 982-600 University Avenue, Toronto, ON, Canada M5G 1X5; ^3^Institute of Health Policy, Management and Evaluation, University of Toronto, 4th Floor, 155 College St., Toronto, ON, Canada M5T 3M6; ^4^Vidya Institute, 253 Christie St., Toronto, ON, Canada M6G 3B8; ^5^Therapeutic Recreation, Sinai Health System-Bridgepoint Site, 1 Bridgepoint Drive, Toronto, ON, Canada M4M 2B5

## Abstract

*Introduction*. The purpose of this study was to evaluate a specialized yoga intervention for inpatients in a rehabilitation and complex continuing care hospital.* Design*. Single-cohort repeated measures design.* Methods*. Participants (*N* = 10) admitted to a rehabilitation and complex continuing care hospital were recruited to participate in a 50–60 min Hatha Yoga class (modified for wheelchair users/seated position) once a week for eight weeks, with assigned homework practice. Questionnaires on pain (pain, pain interference, and pain catastrophizing), psychological variables (depression, anxiety, and experiences with injustice), mindfulness, self-compassion, and spiritual well-being were collected at three intervals: pre-, mid-, and post-intervention.* Results*. Repeated measures ANOVAs revealed a significant main effect of time indicating improvements over the course of the yoga program on the (1) anxiety subscale of the Hospital Anxiety and Depression Scale, *F*(2,18) = 4.74, *p* < .05, and *η*_*p*_^2^ = .35, (2) Self-Compassion Scale-Short Form, *F*(2,18) = 3.71, *p* < .05, and *η*_*p*_^2^ = .29, and (3) Magnification subscale of the Pain Catastrophizing Scale, *F*(2,18) = 3. 66, *p* < .05, and *η*_*p*_^2^ = .29.* Discussion*. The results suggest that an 8-week Hatha Yoga program improves pain-related factors and psychological experiences in individuals admitted to a rehabilitation and complex continuing care hospital.

## 1. Introduction

Yoga is an ancient mind-body practice that is embedded in Vedic traditions dating back to 3000 BC [1] and which is being applied in developed countries as a broad remedy to attenuate health-related symptoms in clinical populations [2, 3] across institutional, community, commercial, and private settings. Yoga is traditionally understood as cultivating concentrative awareness and a unified experience of the self through physical postures (āsana), breathing exercises (prāṇāyāma), inner awareness (pratyāhāra), concentration (dhāraṇā), and meditation (dhyāna), with consequent improved health through a separation process from afflictive cognitive, emotional, behavioural, and autonomic patterns and a shift towards adaptive coping skills [4, 5].

Yoga is garnering attention for its ability to simultaneously address multiple body systems (e.g., circulatory, neuroendocrine, musculoskeletal, respiratory, viscerosomatic, and immunological) through a dynamic and bidirectional process consisting of both top-down and bottom-up constituents and to yield benefits in well-being and symptom reduction [3, 5]. Burgeoning interest in yoga as a therapeutic intervention for a variety of health conditions has resulted in an expansion of research over the past decade, with the volume of publications increasing by threefold with up to 312 randomized controlled studies noted in 2013 [6, 7]. There are a plethora of lineages and schools of yoga that are evaluated in yoga research trials, but the style of yoga (e.g., a*ṣṭ*ā*ṅ*ga, iyengar, and hatha) does not impact the odds of producing positive outcomes for different conditions [8]. Across many of these studies, yoga is extolled for its many benefits. However, the literature is plagued by studies of poor methodological quality and there has been a call for improving the caliber of research in this area [9].

There is evidence that yoga is effective in the treatment of a variety of acute and chronic conditions [9] either as a stand-alone treatment or as an adjuvant therapy, including low back pain [10, 11], arthritis [12], rheumatic disease and fibromyalgia [13, 14], diabetes [15, 16], cancer and related fatigue [17–21], stroke and related disability [22, 23], sleep disorders [24], renal disease [25, 26], hypertension [27, 28], asthma [29, 30], chronic obstructive pulmonary disease (COPD) [31], psychiatric conditions [32], obesity [33], and neurological conditions [34, 35]. Although there is an abundance of research evaluating the impact of yoga on disease-specific symptoms or quality of life for many chronic conditions, to date there has not been one trial evaluating the effects of a yoga intervention on individuals who are receiving care or rehabilitation for complex chronic disease and disability (CCDD).

CCDD is a term that identifies individuals who have been diagnosed with multimorbidities that affect psychological, social, physical, and vocational functioning and require ongoing health care resource utilization [36–38]. Individuals with complex health conditions have been identified as unique in terms of their specific health care needs and health-related experiences [36]. Although the disease combinations reported in multimorbidity are diverse, the most common diagnoses are diabetes, stroke, hypertension, cancer, arthritis, asthma, fractures, the presence of an artificial knee or hip, fatigue, multiple sclerosis, demyelinating diseases of the central nervous system, gonarthrosis, ataxia, COPD, dependence on renal dialysis, malignant neoplasm of breast/prostate, depressive episodes, and pure hypercholesterolemia [39, 40]. Consistent across studies of this population is the severity of the impact of having multiple conditions [41]. Patients with CCDD have an average of five health conditions (comorbidities) [40] and frequently reported pain, weakness, illness-related symptoms, functional challenges (mobility, activities of daily living, equipment devices, etc.), symptoms of anxiety and depression, and disruptions in independence, recreational activities, occupation, social roles, and self-identity.

Multimorbidity has been associated with low socioeconomic status, female gender, and older age in both longitudinal and cross-sectional studies, with prevalence and incidence rates in older age reported at 55% and 12–33%, respectively, and prevalence rates in young-middle age at 11.3–15.4% [42–45]. Multimorbidity is a complex and heterogeneous disease state, with many of the most prevalent conditions being of global concern, and is increasingly becoming the norm rather than the exception, resulting in high health care resource use [45, 46]. In Canada, 42% of total direct medical care expenses are allotted to the treatment of chronic diseases [47], with up to $52,661 per patient spent on average for the last year of life, in part due to inpatient and long-term care costs [48]. Despite the clear priority of the medical community to address the needs of individuals with chronic disease, the orientation of the health care system as an acute-care focused model means that care for individuals with chronic disease is often ineffective, leaving those with many chronic conditions underserviced [49].

A structural shift towards care that is not disease/injury focused but emphasizes addressing many needs at once has been recommended [50, 51]. Understanding the relationships between physical, psychological, and social factors of health in multimorbidity has been identified as necessary for creating effective treatment [38, 52]. A remodeling of chronic disease services has been proposed to create interventions that harmoniously integrate patient-centered and systemic factors and that also target risk factors, such as depression or functional ability, with the end goal of improving patient self-efficacy, functional health status, health-related behaviours, and psychological well-being [49, 53]. Despite an increase in research over the past two decades on multimorbidity, there is still limited research on effective interventions to adequately service this population [53, 54], which highlights a need for programs that are designed to address the needs of individuals who are managing multiple intersecting health impacts of a chronic nature.

Given the evidence supporting the use of yoga for many of the common primary and secondary diagnoses of patients with CCDD (e.g., musculoskeletal conditions, multiple sclerosis, hypertension, arthritis, renal disease, depression, diabetes, COPD, cholesterol levels, and breast and prostate cancer), it is possible that yoga may be able to address many of the multiple health needs these individuals report as being important [40]. Other frequently reported symptoms (e.g., pain, fatigue, emotional upset, nausea, and difficulty breathing) and facets of living that are disrupted (e.g., mobility, activities of living, and social well-being) have also been shown to improve with yoga practice [2, 19, 21, 55, 56]. Moreover, yoga provides a lasting behavioural skill set that increases confidence and self-efficacy and shows maintenance of functional and coping gains in chronic pain patients at follow-up [57]. It can be used in the treatment of chronic conditions for both low- and high-income populations, is associated with treatment adherence in sedentary adults, and holds promise as a cost-effective treatment for chronic conditions [58–61]. Complementary and alternative therapies such as yoga have been recommended for integration into clinical health psychology settings in order to more broadly address well-being, spirituality, multiple health problems, dissatisfaction with orthodox medicine, and disease prevention [62].

Although there is evidence that yoga provides mental and physical health benefits for many of the disease states and psychosocial impacts that are prevalent in individuals with multimorbidity, there have been no studies evaluating its use for this population. Medical rehabilitation and complex continuing care support tend to focus on addressing physical ailments and neglect integrating mental health support. Yoga is an example of a strategy that addresses both; thus it may impart benefits in multiple areas of health, rendering it particularly useful for this population. Hospital and tertiary care settings typically implement evidence-based practice, so there is a need for information regarding the benefits and safety of yoga when used in the treatment of individuals with CCDD or multimorbidity.

This study evaluated the impact of a specialized yoga program on pain, psychological, functional, and spiritual constructs in individuals receiving complex continuing care or medical rehabilitation. Given the complex presentation of impacts for this population, multiple measures were used to fully explore the possible effects of yoga across various aspects of experience. The study used a pilot cohort study design to test the following hypotheses: (1) scores on measures of pain, pain catastrophizing, stress, anxiety, depression, and experiences of injustice will decrease from pre- to post-intervention and (2) scores on mindfulness, self-compassion, and spiritual well-being will increase from pre- to post-intervention.

## 2. Materials and Methods

### 2.1. Participants

In order to be included in the study, participants had to be inpatients at Sinai Health System (Bridgepoint Hospital (BH) Site), be able to understand and speak English, and be cognitively able to understand instructions. Exclusion criteria included a regular yoga practice in the six months prior to the commencement of the study, an expected discharge date before the completion of the yoga program, or moderate cognitive impairment as indicated by a cognitive screen done by BH care team. Participants either were wheelchair users or were comfortable doing yoga from a seated position.

Demographic information and clinical characteristics of the sample are summarized in Table 1. Participants had all been admitted to BH in 2014 and were receiving either complex continuing care (CCC; *n* = 9) or medical rehabilitation (MR; *n* = 1). The one patient that was admitted for MR was informally transferred to CCC partway through the hospital stay for more intensive care. Examination of hospital records across a range of assessment dates indicated that participants had different levels of independence for tasks of daily living and mobility, such as transferring from a bed to a wheelchair. Information recorded within the hospital system is different for the complex continuing care and medical rehabilitation streams.

Both males (*n* = 4) and females (*n* = 6) participated in the yoga program. Height and weight were taken from hospital records for participants receiving CCC and if multiple weight assessments were provided, the weight assessment time closest to the start date of the yoga program was used. Weight and height were taken from self-report data for the participant receiving MR; secondary conditions were not reported for this patient. Participants had been diagnosed with at least one medical condition (see Table 2) and on average 7.6 ± 2.8 conditions. Although most conditions were accompanied by a formal disease diagnosis, some documented conditions were not necessarily accompanied by diagnoses (e.g., weight issues, allergies, pain, and instability). Participant use of various pain treatments (pharmacological, natural health products, physical treatments, psychological treatments, and medical interventions) are displayed in Table 3. The study researchers worked with hospital staff to obtain medical clearance notes for all participants, indicating that it was safe for participants to participate in an eight-week yoga program. Participants did not receive financial compensation for participating in the study.

### 2.2. Procedure

The research protocol was reviewed and approved by the Human Participants Review Committee at York University and by the Joint Bridgepoint Health, West Park Healthcare Centre, Toronto Central Community Care Access Centre (CCAC), and Toronto Grace Health Centre Research Ethics Board.

### 2.3. Research Design

**Figure 1:**
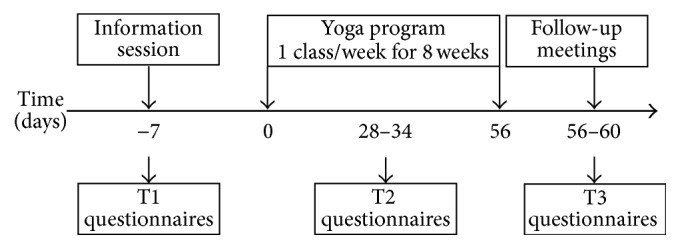
Time sequence of the study intervention. The information session was held seven days before the yoga program began. The yoga program was held once weekly for 8 weeks. Questionnaires measuring pain, pain-related variables, psychological factors, and mindfulness were evaluated at three time points: T1, T2, and T3.

This prospective, pilot study consisted of two parts: a Codesign Phase and a Research Intervention Phase. The Codesign Phase involved consultation with BH staff (Therapeutic Recreation staff, research scientists, the Chair of Complex Chronic Disease Research, the Director of Professional Practice, and a liaison to the research ethics board) to discuss the best approach to the yoga program development, delivery, and evaluation so that it would contribute to meeting the complex needs of the patients. As depicted in Figure 1, the Research Intervention Phase consisted of several components: an information session, yoga classes, follow-up meetings, and administration of self-report questionnaires. Questionnaires concerning pain and related variables, psychological factors, and mindfulness were administered at three time (T) points: pre- (T1), mid- (T2), and postintervention (T3). There were no subsequent data collection points in the time following the yoga intervention. The information session was held seven days before the yoga program began. The yoga program ran for eight weeks (one class/week) and the follow-up meetings took place after the final class and in the following few days. The information session and yoga classes were held in one of two auditoriums at the hospital.

### 2.4. Information Session and Data Collection

Interested individuals were informed about the yoga study by hospital staff (Therapeutic Recreation team members and support staff) and were screened for eligibility. Eligible participants attended the information session (T1) where they were given information about the investigators, the content of the yoga program, expectations for attendance and commitment, possible initial increases in pain due to exertion, and the homework component. Interested individuals had the opportunity to ask questions or voice concerns and those who decided to participate completed the consent process. After written informed consent was obtained, participants were provided with a canvas tote folder, which included an MP3 player with the homework audio files (see yoga program description), a copy of the consent form for their records, and a handwritten instruction guide to using the MP3 player with accompanying illustrations. Participants filled out a form with questions regarding demographic information, health history and current health status, and the questionnaire package. At T2, participants had the option of remaining after the yoga class to fill out questionnaires or taking the questionnaires back to their hospital room to fill out prior to the following class. At T3, participants filled out the questionnaire package after the final class.

### 2.5. The Yoga Program

The specialized yoga program consisted of an integrated approach to Hatha Yoga: postures (āsana), breath awareness exercises (prāṇāyāma), concentrative, meditative, and relaxation practices (dhāraṇā, dhyāna), and yoga philosophy (jñāna) [63–65]. One of the most ancient scriptures of Hatha Yoga outlines yoga as a purificatory practice that balances the activities and processes of the physical body, the mind, and the overall energy level, in order to cultivate health, self-awareness, and inner development [65]. Hatha Yoga was selected as an appropriate form of yoga for individuals with chronic conditions and mobility restrictions as it is gentle and can be easily modified. The yoga philosophy component (see Table 4) was based on relevant contemplative and reflective practices from Patañjali Yoga Sūtras that focused on self-study, personal development, observances, yamas (ethical discipline), and attitudes of acceptance, among others [4, 64, 66]. Concepts found in classical scripture, such as the kośas theory of self, are being integrated in protocols for yoga interventions for chronic illness with a mind-body component [67]. The classes were one-third āsana, one-third relaxation training, and one-third yoga philosophy. The class format, structure, and content were designed in accordance with the yoga literature. A BH Recreation Therapy Assistant was present at each session. All participants practiced from a seated position, using either a wheelchair or a table chair to allow for uniform practice of the āsanas across participants.

Participants were provided with two recordings and were instructed to practice using the MP3 player twice a week. The first recording was a guided body scan awareness practice (~30 minutes) and the second recording was an āsana practice (~15 minutes). The participants were not given the second recording until they were familiar with the yoga āsanas and the teacher decided that they were safe to practice them on their own.

### 2.6. Measures

#### 2.6.1. Brief Pain Inventory-Short Form (BPI-SF) [68]

The BPI-SF is a 9-item self-report questionnaire that measures various aspects of pain and pain interference with daily activities. The Brief Pain Questionnaire [69] and the Brief Pain Inventory [70, 71] were originally developed to evaluate cancer-related pain and have since been validated for other types of pain [72, 73]. In the BPI-SF, individuals are queried on pain history, are asked to visually depict pain locations on a human body diagram, and are asked to indicate best, worst, average, and current pain levels according to 11-point Likert scales, ranging from 0 (*no pain*) to 10 (*pain as bad as you can imagine*). Participants are also queried about pain medications and treatments and the perceived effectiveness of those medications. Finally, individuals respond to items regarding how pain interferes with seven domains of functioning: general activity, mood, walking ability, normal work, relations with other people, sleep, and enjoyment of life, according to 11-point scales ranging from 0 (*does not interfere*) to 10 (*completely interferes*).

The BPI has strong internal consistency (Cronbach's *α* = .85 and .88 for the intensity and interference scales, resp.), adequate construct validity (scores on the interference scale correlate with other pain disability measures) and is sensitive to treatment [70]. The BPI-interference items have been used in studies evaluating pain in individuals with SCI [74, 75] and have been recommended for use in this population, though item 9c, which refers to “Walking Ability,” should be changed to “Ability to Get Around” [76]. It has excellent internal consistency (*α* > .90) and is positively associated with pain intensity (*r* > .60) [77].

#### 2.6.2. Pain Catastrophizing Scale (PCS) [78]

The PCS is a 13-item self-report questionnaire that measures catastrophic thinking in relation to experienced or anticipated pain. Participants are asked to read each item and indicate the extent to which they experience certain thoughts and feelings when experiencing pain by selecting a number from 0 (*not at all*) to 4 (*all the time*). Scores range from 0 to 52, with higher scores reflecting higher levels of pain catastrophizing. The PCS yields a total score and three subscale scores assessing rumination (focus on pain sensations), magnification (exaggerating the threat value of pain sensations), and helplessness (perceiving oneself as unable to cope with pain symptoms). The PCS has high internal consistency (coefficient *α*: total PCS = .87, rumination = .87, magnification = .66, and helplessness = .78) [78].

#### 2.6.3. Perceived Stress Scale (PSS) [79]

The PSS is a 10-item self-report questionnaire that measures symptoms of stress over the past month, in relation to life events and relationships. Participants indicate how much they are experiencing their life as unpredictable and uncontrollable and how much they have felt overloaded. Each item is rated on a 5-point scale, ranging from 0 (*never*) to 4 (*very often*), with a highest possible score of 40, such that higher scores are indicative of higher levels of stress. It correlates well with other measures of stress, such as life events, and depression and anxiety scales and has satisfactory internal reliability (*α* = .78–.82) and test-retest reliability (*r* = .55–.85) [79, 80].

#### 2.6.4. Hospital Anxiety and Depression Scale (HADS) [81]

The HADS is a 14-item self-report questionnaire that measures symptoms of anxiety (7 items) and depression (7 items). For each item, participants are asked to select one from among four possible choices (scored from 0 to 3) that best describes how they have been feeling over the past week. The HADS yields an anxiety (HADS-A) and a depression (HADS-D) subscale score, each with a maximum total score of 21, where higher scores indicate higher levels of anxiety and depression. Scores of 8–10 are considered cut-off points that are clinically meaningful for symptoms of anxiety and depression [81]. Internal consistency is high for both the HADS-A (*α* = .83) and HADS-D (*α* = .82) subscales [82]. Concurrent validity of the HADS is very good, as measured by correlation coefficients of between .62 and .73 for the HADS-D with various well-validated depression scales and correlation coefficients of between .49 and .81 for the HADS-A with various well-validated anxiety measures [82].

#### 2.6.5. Injustice Experiences Questionnaire (IEQ) [83]

The IEQ is a 12-item questionnaire that evaluates feelings and thoughts of perceived injustice and severity of loss in relation to injury or pain. This scale is designed to evaluate cognitive appraisals that contribute to pain-related occupational disability. Individuals answer each item using a 5-point scale, ranging from 0 (*never*) to 4 (*all the time*). This scale has a total score and two subscales, blame/unfairness and severity/irreparability of loss. The total scale has good internal consistency (*α* = .92) and all items correlated above .05 with the total score [83]. This scale has been correlated with pain severity, pain catastrophizing, fear of movement, perceived disability, and depression (*r* = .54–.75, *p* < .01), indicating good construct validity. Cross-sectional regression analysis has shown good discriminant validity in that IEQ contributes to the variance of the predication of pain severity (*β* = .44, *p* < .05) [83]. Test-retest variability of the IEQ is good and scores across time are stable (*r* = .90, *p* < .01); authors note that the test-retest scores were more stable than scores on measures of pain and related constructs (e.g., PCS, Pain Disability Index, and McGill Pain Questionnaire) [83].

#### 2.6.6. Five-Facet Mindfulness Questionnaire-Short Form (FFMQ-SF) [84]

The FFMQ-SF is a 24-item version of the original 39-item FFMQ and has been validated in individuals with depression, anxiety, and fibromyalgia [84]. It is a self-report questionnaire that measures levels of mindfulness according to five facets, which have acceptable model fit with the five-factor structure of the FFMQ. Those facets are observing, describing, acting with awareness, nonjudging of inner experience, and nonreactivity to inner experience. Participants respond to each item by selecting the number that is “most generally true” of his/her experience, on a scale of 1 (*never or rarely true*) to 5 (*very often or always true*). Total scores range from 0 to 120 and higher scores indicate greater levels of mindfulness. The FFMQ is based on a factor analytic study of five independently developed mindfulness questionnaires, with good internal consistency and construct validity [85, 86]. Total facet scores of the FFMQ-SF are highly correlated with the original version, *r* = .89, .89, .92, .96, and .95, for observing, describing, acting with awareness, nonjudging, and nonreactivity, respectively [84]. The correlation alphas are all above the defined criterion of .7 and all intercorrelations between facets and with other constructs are very virtually the same as the FFMQ. All the facets of the FFMQ*-*SF are sensitive to change and had moderate-large effect sizes [84].

#### 2.6.7. Self-Compassion Scale-Short Form (SCS-SF) [87]

The SCS-SF is a self-report 12-item version of the original 26-item questionnaire [88] that measures levels of self-compassion. Self-compassion, as measured by this scale, is defined as the ability to hold one's feelings of suffering with a sense of warmth, security, or concern [87]. This short form has been demonstrated to have a unidimensional construct of self-compassion and also a multidimensional construct consisting of 6 subscales including self-kindness, self-judgement, common humanity, isolation, mindfulness, and overidentified; however, it is not recommended to use subscales for the short form version. The questionnaire queries respondents to indicate “how I typically act towards myself in difficult times,” according to a scale of 1 (*almost never*) to 5 (*almost always*). This scale has been shown to have adequate internal consistency (Cronbach's *α* ≥ .86 for three different samples) and good correlation with the full version (*r* ≥ .97 for three samples). The total score for the short form is calculated by dividing the total score by 12 (for each item) to produce a mean score (personal communication with Kristen Neff, April 19, 2016).

#### 2.6.8. Functional Assessment of Chronic Illness Therapy-Spiritual Well-Being (FACIT-SpWB) [89]

The FACIT-SpWB is a 12-item self-report questionnaire that evaluates experiences of spirituality in individuals with chronic illnesses. The original scale has a total score and two subscales: meaning/peace and faith, although confirmatory factor analysis has validated the three-factor model in which meaning and peace are unique subscales [90]. Questions query themes of harmony and peacefulness and a sense of strength and comfort in one's beliefs. Participants answer each item using 5-point Likert scale, from 0 (*not at all*) to 4 (*very much*). This scale has good internal consistency for the overall index and for the two subscales (*α* = .81–.88). This scale also shows good validity; both the total scale and each subscales were positively correlated with measures of quality of life in cancer patients (Functional Assessment of Cancer Therapy-General) and negatively with measure of mood (Profile of Mood States) [89].

### 2.7. Data Analysis

Statistical Analysis was performed using SPSS Version 23 and SAS Version 94. Exploratory analysis was conducted to evaluate missing data and assumptions of normality. Raw data were evaluated for skew and kurtosis. All self-report measures were assessed for normality using the Shapiro-Wilk test. The self-report data were analyzed using repeated measures ANOVAs (T1, T2, and T3) and Bonferroni post hoc analysis in the presence of a significant main effect of time. Sphericity was evaluated using Mauchly's Test of Sphericity and, in the case of violations, Huynh-Feldt adjustments were used. Simple mediation analysis was conducted using a bootstrapping approach (2,000 resamples), as recommended for small sample sizes which may have violations of normality [91], to evaluate the mediating effect of total SCS scores at T2 on the relationship between HADS-A scores at T1 and T3.

## 3. Results

### 3.1. Preparation of Data

Data were analyzed by a protocol compliance (PC; *n* = 6) and intention-to-treat approach (ITT; *N* = 10). Although one of the participants had missed yoga classes 3 and 4 due to medical reasons, data were collected for this participant at T2 as they had not formally withdrawn from the study by that time. Therefore, for ITT analysis, data were carried forward from T1 for two participants and from T2 for two participants. One participant did not fill out the HADS questionnaire at T1, so T2 scores for this participant were used as a baseline score.

Data were explored for assumptions of normality. Values of kurtosis and skewness for all total scale scores at each time point were converted to *z*-scores for both PC and ITT data. At T1, all were within normal limits (<|1.96| at *p* < .05) except BPI-3 (significant skew). For PC data, all were within normal limits (<|1.96| at *p* < .05) except BPI-3 at T3 (significant kurtosis and skew) and BPI-5 at T3 (significant skew). For ITT data, all were within normal limits (<|1.96| at *p* < .05) except BPI-3 at T3 (and significant skew) and SCS-SF at T3 (significant skew). Similarly, the Shapiro-Wilk test revealed that all total scale scores were normal at *p* < .05, with the exception of violations of normality for BPI-3, *W*(10) = .81, *p* < .05, and BPI-6 at T1, *W*(10) = .84, *p* < .05, BPI-3-PC at T2, *W*(8) = .80, *p* < .05, BPI-3-PC at T3, *W*(5) = .55, *p* < .05, BPI-3-ITT at T3, *W*(10) = .81, *p* < .05, BPI-4-PC at T3, *W*(5) = .75, *p* < .05, BPI-4-ITT at T3, *W*(10) = .84, *p* < .05, BPI-5-ITT at T3, *W*(10) = .81, *p* < .05, and SCS-SF-total-ITT at T3, *W*(10) = .74, *p* < .05.  Table 5 shows the means and sds for each measure across the three time points, as well as significant *p* values and effect sizes.

### 3.2. Demographic and Clinical Variables

**Figure 2:**
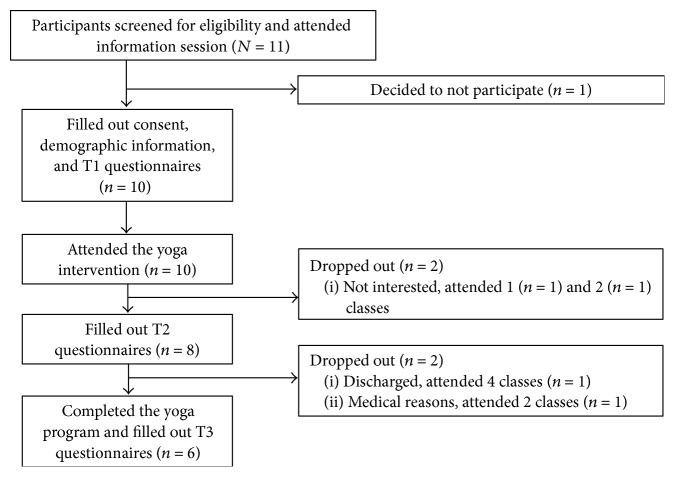
Participant flow through the study.

Figure 2 shows the flow of participants through the study, which ran from October 28 to December 16, 2014. Eleven participants were recruited by hospital staff and attended the information session, 10 of whom provided consent, filled out T1 questionnaires, and participated in at least one class of the yoga program. One participant decided not to participate after learning more about the questionnaire component of the research study. Data for eight and six participants were obtained at T2 and T3, respectively.

### 3.3. Yoga Program Attendance

Of the 10 participants who started the yoga program, six (60%) completed it. Three participants attended 1-2 classes and withdrew for personal or medical reasons, while one participant attended 4 classes, after which she withdrew as she was discharged early from the hospital. The mean ± sd number of yoga classes attended for all participants who entered the program (*N* = 10) was 3.72 ± 2.54 (out of 8 classes) and the mean ± sd number of yoga classes attended for all participants who completed the program (*n* = 6) was 6.83 ± 0.75 (out of 8). The mean ± sd number of participants who did some homework each week (listened to a recording 1–4 times) was 4.0 ± 1.83.

### 3.4. Treatment Results

#### 3.4.1. Pain and Related Variables, Psychological Factors, and Mindfulness

**Figure 3:**
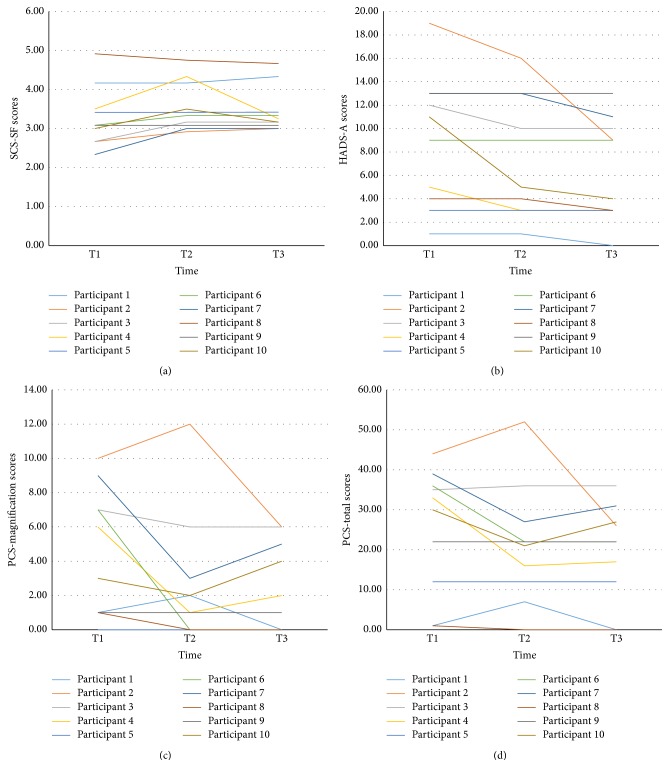
(a) Individual participant Self-Compassion Scale-SF (SCS-SF) scores by time. (b) Individual participant Hospital Anxiety and Depression Scale-Anxiety (HADS-A) scores by time. (c) Individual participant Pain Catastrophizing Scale (PCS)-magnification subscale scores by time. (d) Individual participant Pain Catastrophizing Scale (PCS)-total scores by time.

Repeated measures ANOVAs did not reveal significant changes in any variable across time for the PC analyses. All analyses are reported according to the ITT principle as outlined above. Repeated measures ANOVAs revealed a significant main effect of time for HADS-A, *F*(2,18) = 4.74, *p* < .05, and *η*_*p*_^2^ = .35, for SCS-SF-total (Greenhouse-Geisser adjusted *F*-test), *F*(2,18) = 3.71, *p* < .05, and *η*_*p*_^2^ = .29, and for PCS-magnification, *F*(2,18) = 3.66, *p* < .05, and *η*_*p*_^2^ = .29. Bonferroni comparisons revealed a trend for improvement from T1 to T2 for SCS-SF-total, *p* < .07, and for PCS-magnification from T1 to T3, *p* = .08. A repeated measures ANOVA also revealed a trend of improvement for main effects of time for PCS-total, *F*(2,18) = 2.63, *p* = .099, and *η*_*p*_^2^ = .23. Individual score trajectories for scales with significant changes or trends for improvement are shown in Figure 3.

In terms of clinically meaningful cut-off points, 6 participants had scores above 8 on the HADS-A subscale at T1 and 4 participants had scores at or above 8 on the HADS-D subscale at T1. Out of the six true completers at the end of the study, there were 2 participants with scores above 8 on the HADS-A subscale and 1 participant with a score above 8 for the HADS-D subscale. Using the ITT scores for all participants, there were 5 participants with scores above 8 on the HADS-A subscale and 2 participants with a score above 8 for the HADS-D subscale. Visual inspection of the ITT data revealed that each participant's score for HADS-A and HADS-D remained the same or decreased with the exception of 1 participant, whose score increased 2 points from T1 to T3 on HADS-D.

#### 3.4.2. Mediation Analysis

Nonparametric bootstrapping analysis showed that the total effect of HADS-A scores at baseline on HADS-A scores at the end of the intervention was significantly reduced when SCS-SF scores at midintervention (the mediator) were added to the model (mean = 0.35, SEM = 0.33; CI_.95_ = 0.05, 1.41). As such, the true indirect effect is estimated to lie between .05 and 1.41 with 95% confidence; as zero is not within the CI interval, it can be concluded that the indirect effect is significantly different than zero, *p* < .05, and that mid-treatment SCS-SF scores mediated the relationship between baseline and end-of-treatment HADS-A scores.

## 4. Discussion

This pilot study is the first reported trial to evaluate the effects of a yoga intervention on pain and related variables, psychological constructs, spirituality, and mindfulness in a sample of inpatients receiving complex continuing care/rehabilitation for multimorbidities. The results demonstrate post-intervention improvements in anxiety symptoms, the magnification aspect of pain catastrophizing, and self-compassion. As well, self-compassion was found to mediate improvements in anxiety from pre- to postintervention. These results suggest that a Hatha Yoga program specifically tailored to the needs of a hospitalized population experiencing multimorbidities may provide some psychological benefits.

The finding that anxiety scores were significantly lower after the eight-week program is consistent with RCTs that demonstrated improvements in anxiety and health outcomes in individuals with chronic diseases (diabetes or chronic low back pain) who participated in a yoga intervention when compared to walking or exercise/counselling control groups [92, 93]. A recent cross-sectional assessment of a large sample of individuals with a range of chronic illnesses found that self-reported duration of practice (practice session length and number of months practicing) predicted anxiety and the authors concluded that increased doses of yoga practice may help individuals respond to illness with lower levels of anxiety [94]. As well, systematic reviews document improvements in anxiety for several health populations that have participated in a yoga intervention, such as cancer, stroke, and irritable bowel syndrome [19, 31, 95]. In addition, low-income or noninsured individuals who participated in an integrated program involving mindfulness, self-compassion, and yoga, according to a single-group, repeated measures design, were found to have lower levels of anxiety and depression after intervention [96]. It is apparent that yoga provides psychological symptom improvement in both health populations and those who are impacted by low health care resources. This combination of experiences (health concerns, anxiety symptoms, financial strain, and a lack of resources) parallels the presentation of individuals with CCDD and indicates that yoga may reduce anxiety in the context of multiple health-related impacts.

Although pain catastrophizing has been less well studied in yoga trials, two studies (one pilot, one RCT) found that levels of pain catastrophizing were reduced from pre- to post-yoga interventions in samples of women with fibromyalgia [97, 98]. The present results are consistent with these studies in that we found the magnification aspect of pain catastrophizing (e.g., “I wonder whether something serious might happen”) decreased from pre- to post-intervention. Pain catastrophizing is a strong predictor of pain severity, pain-related interference, disability, depression, and altered social support networks [99] and is associated with physical function deterioration in individuals with joint pain and comorbidity [100], highlighting it as a useful target for interventions that intend to increase functional ability in individuals with multimorbidity or CCDD. Other pain-related psychosocial factors, such as pain disability and pain acceptance, have been shown to improve with yoga practice [101]. Taken together, these findings provide some evidence that yoga may help to reduce the threat value attributed to pain stimuli or alter pain-related experiences in individuals with medical conditions in which pain is a predominant feature. As well, the potentially debilitating impact of pain-related disability or chronic health stress on financial and social independence for individuals with CCDD may amplify magnification cognitions, pointing to the utility of targeting this construct in yoga interventions.

The benefits of yoga extend beyond decreasing negative cognitive-affective experiences and can also serve to generate or augment a nurturing, positive, and discriminative approach to engaging with inner experiences. Self-compassion is a Buddhist concept that is increasingly being considered as an important mental health construct in Western Psychology and entails three main components: self-kindness, common humanity, and mindfulness [102]. The present findings that self-compassion increased significantly from pre- to post-intervention parallel the results from a yoga research trial in individuals living with an implantable cardioverter defibrillator [103]. The results of that study showed that participants who were randomized to a once weekly, eight-week yoga intervention reported increased self-compassion at the end of the trial compared to a usual care group [103]. For individuals with severe health impacts who typically use avoidance or distancing as coping strategies, a yoga practice may enable them to contact suffering and pain without judgmental or comparative thoughts [102]. Yoga philosophy didactics, which explain that inadequacies, failings, and suffering are considered part of the human condition (shared humanity), may normalize challenging experiences and enable individuals with CCDD to extend forgiveness towards their own short-comings and pain, rather than orienting from the stigma and marginalization that can accompany disability.

### 4.1. Mechanisms of Action

The finding that self-compassion mediated decreases in anxiety provides some support for self-compassion as a protective agent in distressing psychological experience and in helping to understand how yoga may be exerting its mechanism of action. This construct has been identified in the yoga literature as one of seven possible mediators of yoga and stress; other mediators include psychological (positive self-affect and mindfulness) and biological (activity in the posterior hypothalamus and inflammatory and endocrine responses: C-reactive protein, Interleukin-6, and cortisol) pathways for therapeutic effects [104]. This is the first study to date that demonstrated the mediating role of self-compassion on psychological changes in a population experiencing medical concerns who participate in a yoga program. There is one previous trial that has demonstrated self-compassion and mindfulness as mediators of quality of life and stress in healthy young adults who participated in a four-month residential yoga intensive [105]. By contrast, self-compassion and mindful attention were not found to mediate changes in emotional stability in high school students who participated in a 16-week yoga program, when compared to students who participated in physical education as usual [106].

This construct may be more amenable to facilitating secondary mental health benefits in individuals experiencing illness-related duress and may impact how individuals cope with chronic and debilitating illnesses. It has been shown to change emotional responses, such as shame, and increase positive coping behaviours in individuals living with HIV and it predicts positive attitudes in the elderly, potentially serving as a buffer against the inevitable challenges associated with age decline [107, 108]. In addition, it is positively associated with both intentions to engage with and practice of health-promoting behaviours (e.g., eating habits, stress management, exercise, and sleep) with indirect effects through adaptive emotions (e.g., health self-efficacy, and positive affect), in community samples of Canadian adults [109, 110]. As the risk of multimorbidity increases with age, augmenting adaptive emotional responses to illness and health-promoting behaviours may assist in the prevention of further health decline and the promotion of well-being.

Although mindfulness was not a mediator of positive psychological change in the present study, previous yoga trials have reported that mid-intervention levels of mindfulness mediated changes in pain catastrophizing from pre- to postyoga intervention in women with fibromyalgia [97]. It may be that these constructs work by exerting different mechanisms during a yoga practice or that one may be more potent as a mediator for different populations or types of mindfulness or yoga interventions. Self-compassion has been demonstrated to be a more robust predictor of symptom severity (e.g., anxious and depressive symptoms) and quality of life than mindfulness in a large community sample of individuals seeking self-help for anxious distress and predicts emotional well-being more consistently than mindfulness in a sample of youth participating in a 5-day meditation retreat [111, 112]. Further examination between psychological well-being, mindfulness, self-compassion, and disability for individuals with CCDD in the context of a yoga intervention is warranted.

### 4.2. Attending to the Signals of the Body: New Pathways

The role of interoception, which is a complex and multimodal bodily system involving a sense of body parts in space (proprioception) and the act of attending, appraising, and responding to afferent body signals [113], has been considered as one of yoga's underlying mechanisms of action through the process of interoceptive exposure and reconditioning [97]. It is proposed that mind-body interventions, such as yoga, may interrupt habitual ways of perceiving and interpreting body sensations within the context of higher-order cognitive processes, such as goals and intentions, by connecting an individual with the present moment and with their agency for personal change [113]. In addition, it has been proposed that higher level brain networks that are activated by yoga practice may serve to inhibit negative appraisals, rumination, and emotional reactivity while lower level neural networks may downregulate physiological responses to stress, such as inflammatory markers and vasopulminary restriction, through the activation of the parasympathetic nervous system [5]. This is relevant in interpreting the results that pain catastrophizing decreased from pre- to post-interventions, as this construct involves exaggerated or negative cognitive-emotional appraisal of painful stimuli as threatening, is accompanied by perceived helplessness (lack of power), and is associated with aberrant central nervous system processes, such as cytokine or hypothalamic-pituitary-adrenal responses to pain, and activation of neural regions involved in processing affective components of pain [99]. It is clear that pain catastrophizing is a construct involving both emotional and biological processes and it may be that yoga helps individuals to reinterpret physical body signals for what they are, rather than as dangerous threats, through two elements of practice, practicing witness consciousness and then actively creating positive change in the body, which is then reinforced with practice through operant conditioning.

Self-compassion mirrors these two elements of traditional yoga practice, in that it involves a truthful recognition of one's inner state and selecting responses or behaviours that will alleviate suffering. It may be that, through yoga, individuals discover safety in opening to distressing experiences, circumventing, or offering a protective buffer against engrained ruminative or punitive “self-talk,” and thereby create new ways of relating to self and prioritizing actions that are consistent with well-being and values. Higher levels of self-compassion have been associated with lower levels of catastrophizing, avoidance, and rumination in chronic pain patients who were presented with vignettes involving a violation of social contract and have been found to predict affect, pain disability, and pain catastrophizing in obese patients with chronic pain [114, 115]. The relationships between self-compassion, anxiety, and disability have been explored in individuals with Generalized Anxiety Disorder; these individuals display lower levels of self-compassion and mindfulness than healthy stressed controls and mindfulness was a better predictor of disability than actual anxiety symptoms, drawing potential protective effects of mindfulness on disability in individuals with chronic worry and physiological symptoms [116]. The relationship between self-compassion and anxiety should be further elucidated and it may be useful to examine the relationships between the subscales of the SCS to better understand which components of self-compassion are most helpful in mediating changes in anxiety in clinical populations.

### 4.3. Limitations

There are limitations to the present study. The primary weakness is the absence of a control group, which is a shortcoming that is widespread in the yoga research literature, and makes it impossible to attribute the improvements observed to the yoga practice itself. As well, the small sample size limits power and introduces the possibility of type II error. Logistical limitations included participant difficulty in using the MP3 players and, as a result, reducing homework engagement and completion, which may have reduced overall efficacy of the eight-week yoga intervention. The difficulties that these patients experienced when using the technology are consistent with a previous report indicating that this population has challenges in paying attention and in using assistive technology [117]. Although the research team carefully selected devices with few buttons and minimal steps required to turn on and navigate the devices and also provided large print diagram instructions to accompany the devices, the population still experienced difficulty, which illustrates that they may be better serviced by yoga interventions that do not involve assistive technology for homework components.

### 4.4. Future Research

The researchers hope that these findings will be considered in the design and implementation of future research projects for individuals who are experiencing CCDD and associated pain, limited mobility, loss of functional ability, severe health impacts, and psychological sequelae. Future research trials should use a randomized, controlled trial study design with appropriate control conditions (e.g., wait-list, exercise, walking, or education) and a longitudinal design with follow-up intervals to determine lasting effects of a yoga practice [118]. Targeting self-compassion in the content and philosophy portions of the yoga interventions may enable researchers to further explore its mediating role of this construct on other psychological or physical experiences prevalent in this population. Trials that seek to further illuminate processes that underlie therapeutic gains may wish to use measures of self-regulation, self-compassion, stress, and positive affect alongside neuroendocrine-inflammatory markers of physiological status [104]. Evaluation of how these variables interact with pain-related constructs associated with the fear-avoidance model of chronic pain (e.g., chronic pain acceptance, pain-related disability, fear of pain, pain anxiety, and pain self-efficacy) may be warranted to better understand the converging impacts that result in distress and disability and with the end purpose to improve health and well-being.

## 5. Conclusions

The results of the present pilot project suggest that an eight-week specialized yoga program may help to reduce anxiety and the magnification component of pain catastrophizing and to increase self-compassion in patients with multimorbidity. This study provides preliminary evidence for yoga as an auxiliary care service that may be amenable to institutions that are in the process of evolving from single-disease treatment frameworks and that are seeking to assimilate programs and services that can address multiple, intersecting health concerns for various ages. The use of a randomized, controlled trial with a larger sample size and a more intensive yoga intervention design (e.g., two or more classes a week for 10–12 weeks) is recommended to further explore the relationships among pain, psychological experience, and mindfulness or spiritual constructs in individuals who are severely impacted by disease and disability.

## Figures and Tables

**Table 1:** Demographics of the sample (*N* = 10).

Demographic	*N* (%)/*M* (sd)
Age (years)	63.1 (16.6)
Height (cm)	163.6 (15.4)
Weight (kg)	70.7 (17.1)
Race/ethnicity	
African Canadian	2 (20%)
European (Italian/Croatian)	2 (20%)
Caucasian	6 (60%)
Socioeconomic class	
High	1 (10%)
Middle-high	1 (10%)
Middle	1 (10%)
Middle-low	2 (20%)
Low	5 (50%)
Level of education (*n* = 9)	
Grade school	2 (22.2%)
High school	2 (22.2%)
University/college	5 (55.6%)
Postgraduate school	0 (0%)

**Table 2:** Primary and secondary conditions by participant (*N* = 10).

Participant	Primary diagnoses	Secondary diagnoses
1	Multiple sclerosis	Instability (report of having fallen).

2	End stage renal disease	Diabetes, hypertension, ischemic disease (unspecified), gastrointestinal issues, renal failure, moderate pain (less than daily), general instability (report of having fallen).

3	Klippel-Feil syndrome	Asthma, emphysema, moderate pain (daily), general instability (report of having fallen).

4	Superficial injury	Hypotension, cerebrodisease, arthritis, Parkinson's disease, asthma, moderate pain (both daily and less than daily), general instability (report of having fallen).

5	Cervical spondylosis	Osteoporosis, hemiplegia, anxiety, allergies, anemia, gastrointestinal issues, pneumonia, moderate pain (daily), general and acute instability (report of having fallen), skin issues (pressure ulcers, rash).

6	Intracranial hemorrhage (NOS, nontraumatic)	Hypertension, cardiovascular disease, aphasia, cerebrodisease, hemiplegia, allergies, pneumonia, urinary tract infection, weight issue, edema, moderate pain (daily), general and acute instability.

7	Hyperkalemia	Diabetes, arthritis, pneumonia, moderate pain (daily), report of having falling.

8	Neuromuscular bladder dysfunction (NOS)	Hypothyroidism, sclerosis (type not indicated), depression, mild pain (daily), general instability, skin issues (pressure ulcers, rash, desensitized skin).

9	Syncope and collapse	Hypertension, osteoporosis, depression, emphysema, gastrointestinal issues, moderate pain (less than daily), report of having fallen, anxiety.

10	Neuromyelitis optica/Devic's disease	Hemiplegia, sclerosis, depression, gastrointestinal, urinary tract infection, moderate pain (daily), general instability, skin issues (rash).

**Table 3:** Pain medications and pain treatments previously or currently used (*N* = 7^*∗*^).

Pain medications and treatments	*N* (%)	Participant number
Pharmacological medications (e.g., opioid-based medications, acetaminophen, and antidepressants)	7 (100%)	1, 3, 4, 5, 7, 8, 10
Natural health products (e.g., supplements and vitamins)	5 (71.43%)	1, 3, 4, 5, 8
Physical treatments (e.g., massage, acupuncture, physiotherapy, and exercise)	5 (71.43%)	1, 3, 4, 5,8
Psychological treatments (e.g., meditation, psychotherapy, distraction, and relaxation)	3 (42.86%)	3, 4, 8

*Note*. ^*∗*^Three participants did not record the use of pain medications or treatments.

**Table 4:** Yoga philosophy concepts by class.

Class number	Concept	Explanation
1	Witness consciousness and ahimsā (nonviolence); Sūtra 2.35.	Practicing “being with” challenging experiences without pushing them away or clinging to personal narratives. Practicing in a way that is safe and supportive.

2	Satya (truthfulness); Sūtra 2.36.	Honestly examining one's experience to better understand one's “starting point” and using yoga practice as a springboard for positive change.

3	Breath awareness to balance the nervous system and calm the mind; Sūtra 1.34.	Pain management through relaxation, training the attention to see tension patterns in the body, and using imagery and visualization.

4	Sthira sukham āsanam; Sūtra 2.46.	Finding a balance between steadiness/stability/effort with ease/joy/relaxation.

5	Ekā gra (one pointed concentration); Sūtra 1.32.	Training attention and concentration by returning to a point of focus repeatedly.

6	Contemplation on the heart; Sūtra 1.36.	The heart as a resource, refuge, and source of inner luminosity.

7	Contemplation of kośas (sheaths/layers).	Five sheaths of the self: physical (annamaya), breath (prāṇāmaya), mind (manomaya), wisdom (vijñānamaya), and joy (ānandamaya). Practicing experiencing parts of the self without identifying with them.

8	Śavasana and the kośas.	Consolidation of all concepts. Cultivation of awareness of the layers of the self and a deeper part that can rest back and witness.

**Table 5:** Mean (sd) values for pain, psychological, and mindfulness variables across time, using Intent-to-treat sample (*N* = 10).

Measure	Preintervention (T1)	Midintervention (T2)	Postintervention (T3)	Significance (*p* value)
BPI-SF-3	6.90 (3.14)	6.80 (2.66)	6.90 (2.81)	ns
BPI-SF-4	4.50 (3.57)	3.60 (3.20)	2.70 (2.54)	ns
BPI-SF-5	5.20 (3.49)	4.50 (2.95)	5.00 (2.91)	ns
BPI-SF-6	6.80 (3.33)	5.30 (3.71)	4.60 (3.47)	ns
BPI-9-SF-total	29.10 (21.27)	23.70 (16.40)	25.50 (17.82)	ns
PCS-total	25.30 (15.62)	21.30 (14.77)	19.30 (12.19)	.099
PCS-helplessness	11.40 (8.58)	9.20 (8.20)	8.70 (6.45)	ns
PCS-magnification	4.50 (3.72)	2.70 (3.74)	2.40 (2.59)	.047^b^
PCS-rumination	9.40 (5.06)	9.60 (4.70)	8.20 (4.98)	ns
PSS	20.20 (7.83)	19.00 (8.27)	15.70 (8.17)	ns
IEQ-total	23.50 (7.11)	20.10 (9.55)	21.10 (11.49)	ns
IEQ-blame/unfairness	10.20 (4.13)	8.10 (4.46)	8.60 (6.10)	ns
IEQ-severity/irreparability	13.30 (4.35)	12.00 (5.91)	12.50 (6.00)	ns
HADS-A	9.00 (5.64)	7.70 (5.19)	6.50 (4.38)	.022
HADS-D	6.70 (4.99)	5.70 (3.83)	5.80 (4.05)	ns
FFMQ-SF-total	84.40 (7.66)	86.60 (10.05)	87.40 (12.40)	ns
FFMQ-SF-observing	15.70 (2.16)	16.80 (2.20)	16.80 (2.15)	ns
FFMQ-SF-describing	19.50 (2.64)	19.10 (3.87)	19.10 (4.09)	ns
FFMQ-SF-acting with awareness	18.10 (1.79)	19.60 (3.06)	18.60 (4.20)	ns
FFMQ-SF-nonjudging	16.90 (4.18)	16.70 (4.37)	17.10 (5.02)	ns
FFMQ-SF-nonreactivity	14.20 (2.20)	14.40 (3.10)	15.80 (4.87)	ns
SCS-SF	3.28 (0.77)	3.57 (0.63)	3.44 (0.58)	.047^a^
FACIT-SpWB-total	33.80 (8.13)	35.00 (9.01)	36.00 (7.45)	ns
FACIT-SpWB-faith	11.80 (4.57)	11.00 (5.29)	12.00 (5.29)	ns
FACIT-SpWB-meaning	12.70 (2.45)	13.50 (2.84)	12.70 (2.16)	ns
FACIT-SpWB-peace	9.30 (3.62)	10.50 (2.55)	11.30 (2.50)	ns

*Note*. Greenhouse-Geisser adjusted *F*-tests for significant main effects of time were conducted for SCS-SF.

*Note*. BPI-SF: Brief Pain Inventory-Short Form, PCS: Pain Catastrophizing Scale, PSS: Perceived Stress Scale-10 Items, IEQ: Injustice Experiences Questionnaire, HADS-A: Hospital Anxiety and Depression Scale-Anxiety, HADS-D: Hospital Anxiety and Depression Scale-Depression, FFMQ-SF: Five-Facet Mindfulness Questionnaire-Short Form, SCS-SF: Self-Compassion Scale-Short Form, FACIT-SpWB: Functional Assessment of Chronic Illness Therapy-Spiritual Wellbeing.

*Note*. ^a^ *p* < 0.1 for T1 versus T2; ^b^ *p* < 0.1 for T1 versus T3.
